# Horizontal versus vertical scapular free flap reconstructions in total maxillectomies without orbital exenteration—A systematic review and clinical case series

**DOI:** 10.1016/j.jpra.2025.09.006

**Published:** 2025-09-13

**Authors:** Eleonora O.F. Dimovska, Francina W. Cobben, Andreas Thor, Andrés Rodriguez-Lorenzo

**Affiliations:** a Department of Plastic & Maxillofacial Surgery, Uppsala University Hospital, Uppsala, Sweden; b Department of Surgical Sciences, Uppsala University, Uppsala, Sweden; c Department of Plastic & Reconstrutive Surgery, Leiden University Medical Centre, Leiden, Netherlands

**Keywords:** Total maxillectomy, Scapular free flap, orbital floor reconstruction, Alloplastic reconstruction, Total maxillectomy without orbital exenteration, Palato-maxillary reconstruction

## Abstract

**Background:**

The scapular free flap is a recognized reconstructive option for total maxillectomies, however, its optimal orientation and position in reconstructions without orbital exenteration is undetermined. The following study aimed to evaluate horizontal and vertical scapular reconstructions of the orbito-palato-maxillary complex by means of a systematic review of the literature and in a clinical case series.

**Methods:**

A systematic review was performed, including articles from Pubmed, Medline, Embase and Cochrane databases. In parallel, a retrospective review of consecutive clinical cases treated between 2016 and 2022 at a tertiary care center in Sweden was performed. Data was collected on scapular flap orientation and positioning, use of adjunctive orbital floor support (vascularized or alloplastic), soft tissue configuration, and postoperative complications related to ocular and palato-maxillary outcomes.

**Results:**

Eleven studies comprising 44 patients met inclusion criteria. Vertically orientated scapulas (26 patients) were most common and demonstrated fewer complications than horizontally orientated scapulas (*p* = 0.04), particularly when excluding alloplastic material by using an osteotomized fragment to reconstruct the orbital floor (13 patients) (*p* = ≤0.001). Adopting a hybrid approach, combining vascularized bone with alloplastic material for enhanced orbital floor support, did not eliminate ocular or infective complications in a clinical case series.

**Conclusion:**

No universally optimal technique exists for reconstructing the total maxillectomy without orbital exenteration, however, the vertically orientated scapula with an osteotomized fragment for orbital floor reconstruction has the potential to offer a “single flap” solution. The indications for alloplastic adjuncts for orbital floor support remain undetermined.

## Introduction

**Figure 1:**
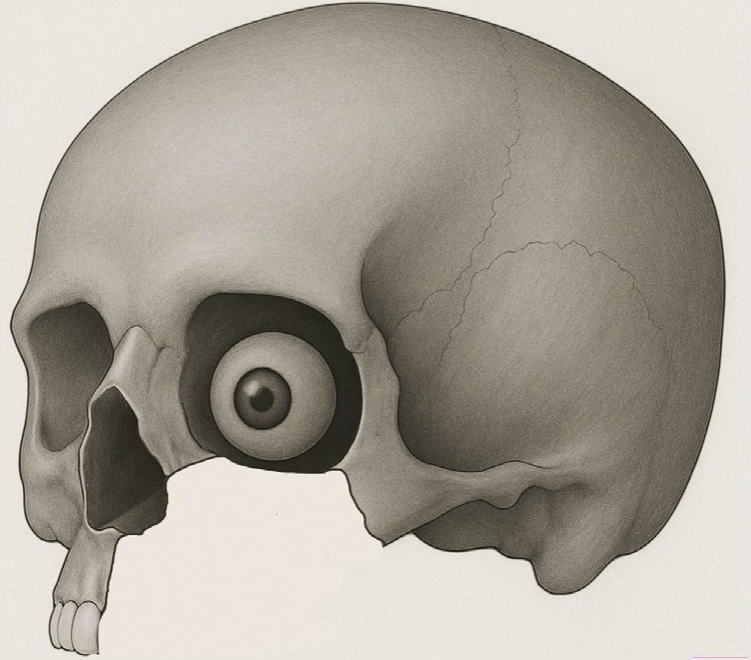
The defect Brown IIIb/Cordeiro IIIa—total maxillectomy without orbital exenteration.

Total maxillectomies without orbital exenteration remains one of the most complex and multifaceted reconstructive challenges in head and neck reconstruction. The defect, a Brown type IIIb or Cordeiro type IIIa (Figure 1), includes the palate with associated dental arch, maxillary sinus, paranasal sinuses, medial orbital wall and orbital floor.^1^^,^^2^ Subsequent reconstruction must address an array of functional and aesthetic demands including restoration of midfacial height and projection, separation of the nasal and oral cavities, dead space obliteration, orbital support and future dental rehabilitation.^3^^,^^4^ Several bone and soft tissue reconstructions have been adopted to address these requirements, often in combination with non-vascularized grafts and alloplastic materials to augment the orbital floor.^3^^,^^5^^,^^6^ However, achieving a simultaneously successful reconstruction of both the palato-maxillary component and orbital floor has proven an arduous task, as many methods succeed in addressing one component, but falls short in the other. With adjuvant radiotherapy being an inevitable must, non-vascularized materials have been cautioned against due to the risk of resorption, infections and extrusion. Consequently, vascularized bone has been advocated as a durable and radiotherapy-resistant option.^4^^,^^5^^,^^7^^,^^8^ Traditionally, the free fibula and iliac crest have been used, however, a high donor site morbidity and complex fibular insets have led to the interest in the scapular free flap; a thin and broad bone with chimeric potential and minimal donor site morbidity.^9^ A critical consideration in maxillary reconstruction is orientation and positioning of the bone. A horizontal palatal position enables optimal palatal conformity and limits oronasal communication, but may compromise orbital support and midface projection. Conversely, a horizontal position at the orbital floor may however compromise the palato-maxillary reconstruction. Vertical orientations offer enhanced midfacial support, but may compromise palatal closure and require additional material for a durable orbital floor reconstruction. Recently, adopting an osteotomy to the vertical scapula has been trialed as a single-flap solution to address both the palato-maxillary component and orbital floor, offering the unique selling point of potentially omitting alloplastic material from the orbital floor altogether. There is however no consensus on the optimal orientation of the scapular free flap to effectively address both the orbital and palato-maxillary components of the total maxillectomy, and on the role of alloplastic materials. Thus, the aim of this study was to evaluate ocular and palatal outcomes in various scapular configurations in the reconstruction of total maxillectomies without orbital exenteration.

## Materials, patients & methods

### Search strategy & eligibility criteria

A systematic literature search was performed using Pubmed, Medline, Embase and Cochrane library databases. An identical search strategy was applied across all platform utilizing the following combination of search terms: (“maxilla” OR “maxillectomy” OR “maxilla resection” OR “maxillary reconstruction” OR “facial” OR “orbit” ‘orbital floor’ OR “head and neck”) AND (“scapular flap” OR “scapula”) AND “reconstruction.” The review was conducted in accordance with the PRISMA (Preferred Reporting Items for Systematic Reviews and Meta-Analyses) guidelines and registered in the PROSPERO (International prospective register of systematic reviews) database (Registration No. CRD42025648700). In parallel, a retrospective review of consecutive clinical cases was conducted at Uppsala University Hospital in Sweden between 2016 and 2022. Ethical approval was obtained from the Swedish Ethical Review Authority (DNR 2022-01,743-01 and 2023-01,847-02). Inclusion criteria encompassed all patients who underwent a total maxillectomy without orbital exenteration. Further eligibility criteria were defined according to the PICOS (participants, intervention, comparator control, main outcome, strategy, and study design) framework (Table 1).

**Table 1:** PICOS framework for study planning.

Letter	Meaning	Inclusion	Exclusion
P	Participants/population	Total maxillectomies (Brown type IIIb or Cordeiro type IIIa) with resection of the orbital floor and preservation of the eye and orbital contents	Orbital exenteration, partial maxillectomies, orbital floor reconstruction-only
I	Intervention(s)	Reconstruction using the free scapular bone flap and/or alloplastic material	Reconstructions using non-scapular bone, pedicled flaps, non-scapular non-vascularized grafts, soft tissue only
C	Comparator(s)/control	None	
O	Main outcome(s)	Ocular (diplopia, enophthalmos, ectropion, lagophthalmos), Palato-maxillary (dental rehab, healing, speech), Other (infection, alloplastic extrusion/dislocation)	Lack of outcome data, unclear orientation of the scapular bone
S	Study design	Randomized controlled trials, nonrandomized trials, cohort studies, case series and case reports.	Radiological studies, general reviews, technique papers, anatomy papers

PICOS, participants, intervention, comparator control, main outcome, strategy, and study design.

### Data collection process and data items

All articles were initially screened by title, followed by abstract if relevant and full-text review when meeting inclusion criteria. From each eligible study, individual patient cases were extracted when sufficient operative and outcome details were available. The literature search and article selection were conducted independently by two authors (EOFD and FC).

Included cases were stratified into six categories based on the orientation and configuration of the scapular free flap**:**

1. Horizontal scapula positioned at the palate with alloplastic orbital floor reconstruction (Figure 2A).

**Figure 2:**
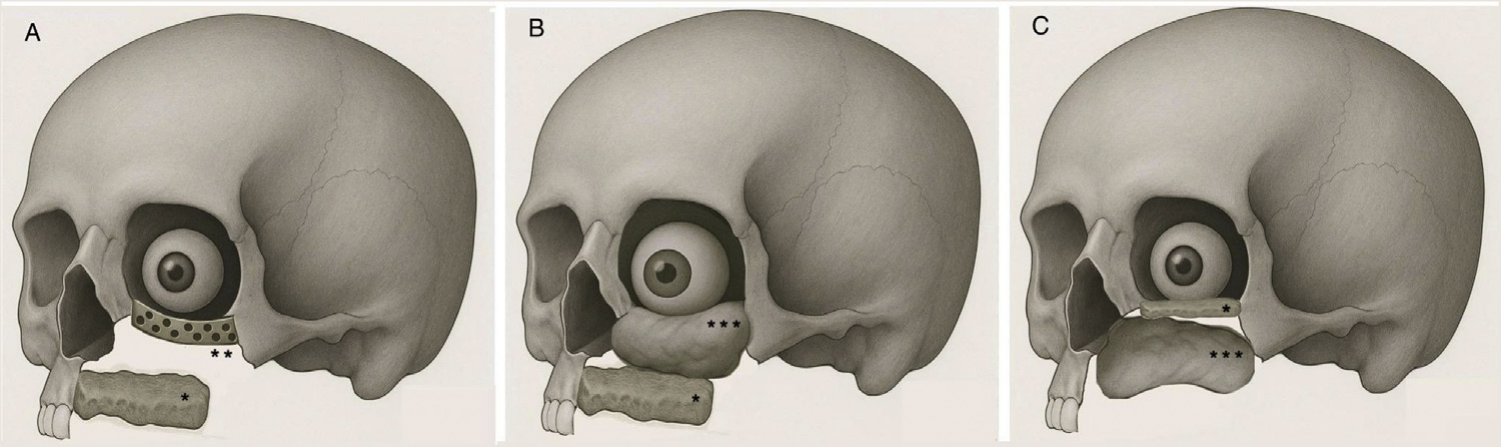
Horizontally orientated scapula (*) at the palate with A. Alloplastic orbital floor reconstruction (**), B. Soft tissue orbital floor reconstruction (***) and C. Horizontal scapula at the orbital floor (*) with soft tissue palatal reconstruction (***).
2. Horizontal scapula positioned at the palate with orbital floor support provided by a de-epithelialized skin paddle (Figure 2B).
3. Horizontal scapula positioned at the orbital floor with inferior palatal reconstruction provided by a de-epithelialized skin paddle (Figure 2C).
4. Vertical scapula for anterior maxillary wall and alloplastic orbital floor reconstruction (Figure 3A).

**Figure 3:**
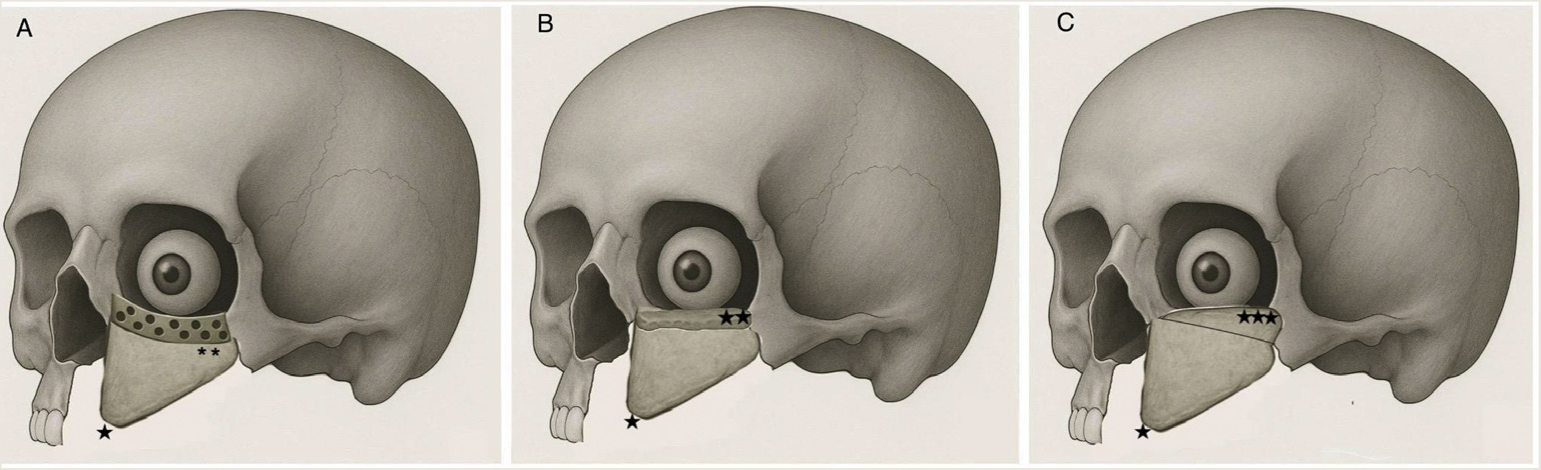
Vertical scapula (★) with; A: Alloplastic orbital floor reconstruction (**), B: Non-vascularized scapular graft orbital floor reconstruction (★★), and C: Vascularized green-stick osteomized scapular fragment orbital floor reconstruction (★★★).
5. Vertical scapula with non-vascularized scapular bone graft used for the orbital floor (Figure 3B).
6. Vertical scapula with a green-stick osteotomized fragment for reconstructing the orbital floor (Figure 3C).

For both the systematic review and clinical case series, data was collected on patient demographics**,** intraoperative flap configuration (including osseous and soft tissue components), skin incision type**,** orbital floor reconstruction method**,** use of virtual surgical planning**,** radiotherapy, and postoperative complications. Primary outcomes included ocular complications (diplopia, enophthalmos, ectropion, lagophthalmos). Secondary outcomes included postoperative infections and complications related to alloplastic materials (extrusion, dislocation) and palato-maxillary outcomes of speech, healing and uptake of dental rehabilitation. Statistical analyses were performed using GraphPad Prism version 10.1.0. Data was considered non-parametric and analyzed using the chi-square test (or Fisher’s exact test for variables ≤5) for nominal variables. A significance level of *p* = 0.05 was set.

### Assessment of study quality and risk of bias

Level of evidence was assessed using the Levels of Evidence for Therapeutic Studies, as defined by the American Society of Plastic Surgeons (ASPS).^10^ Risk of bias was independently assessed by authors EOFD and FC using the ROBINS-I (Risk Of Bias In Non-randomized Studies of Interventions) tool for nonrandomized studies.^11^ It examined seven domains: bias due to confounding, bias in classification of interventions, bias in selection of participants into the study, bias due to deviations from intended interventions, bias due to missing data, bias in measurement of outcomes and bias in selection of the reported result. Each domain was evaluated and judged using signaling questions of: “yes,” “probably yes,” “probably no,” “no” or “no information.” An overall judgement of risk of bias was scored as ”low,” ”moderate” or “high” risk.

## Results

### Systematic review—Search strategy

**Figure 4:**
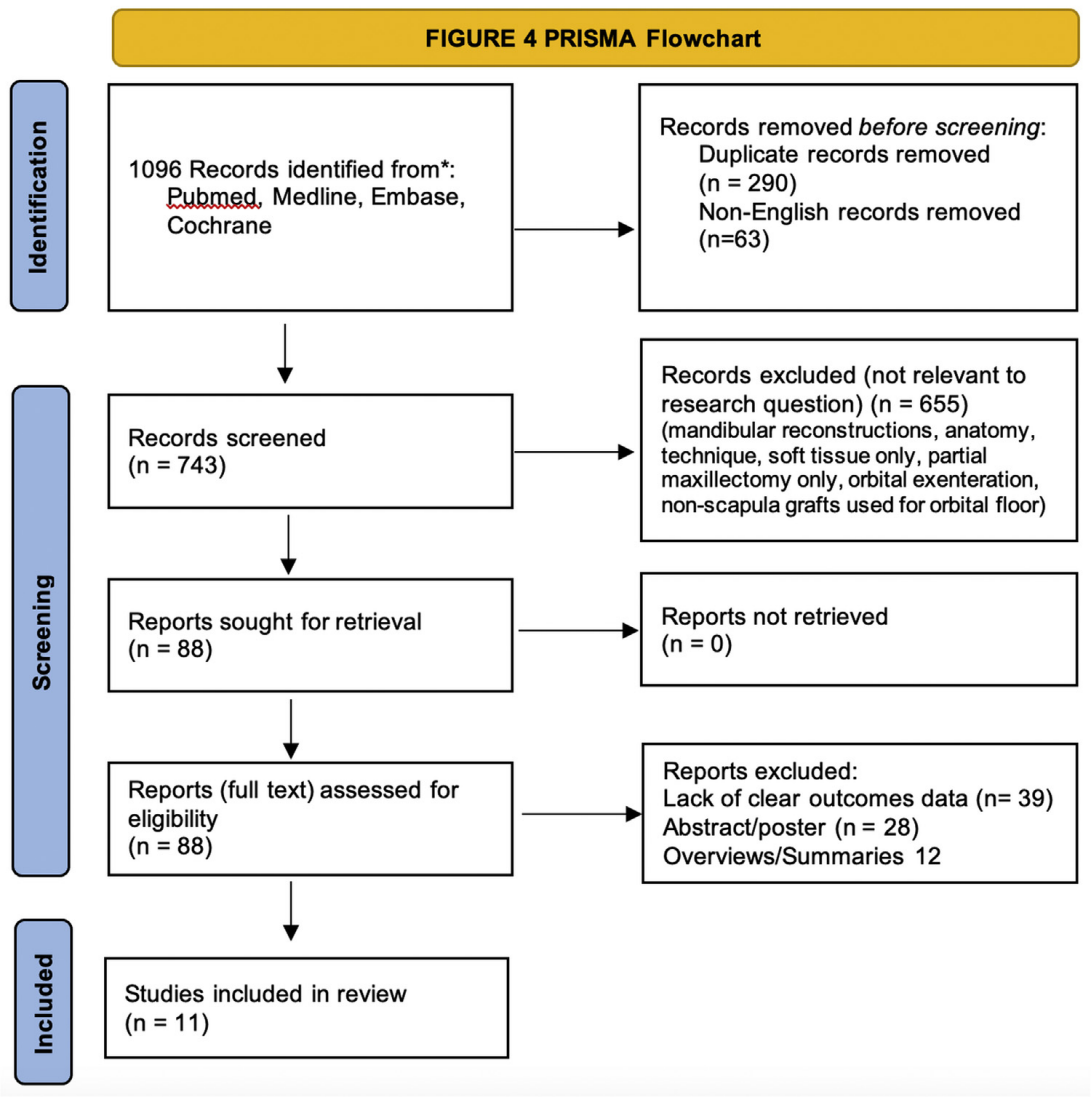
PRISMA Diagram.

An initial database search identified 1096 studies, of which 1008 studies were excluded for duplicate, non-English or non-relevant criteria. A final 88 studies were considered for inclusion of which 76 were excluded due to insufficient clinical outcomes data, donor site focused outcomes, outcomes related to virtual surgical planning, unclear scapular orientation/position or grouped reporting of outcomes for various orientations and/or defects. A final 11 studies met inclusion criteria and are presenting in a PRISMA diagram (Figure 4).

### Systematic review—Study characteristics & results

Among 11 included studies, the scapular free flap was orientated horizontally in five articles (18 patients) (Table 4) and vertically in six articles (26 patients) (Table 2). The horizontal palatal position included three articles and 13 patients of which two articles and 12 patients had alloplastic material as orbital floor reconstruction, and one article with one patient had soft tissue orbital floor support-only in form of a de-epithelialized thoracodorsal artery perforator (TDAP) flap. The horizontal orbital position included three articles with five patients having a de-epithelialized TDAP flap as palato-maxillary obliteration. The vertically orientated scapulas had orbital floor reconstruction with alloplastic material in three articles and six patients, additional scapular graft in one article and two patients and a green-stick osteotomy in three articles and 18 patients. Complications (ocular issues, infections, extrusion) were most prevalent in horizontal scapulas (*p* = ≤0.001), especially when using non-vascularized material such as grafts or alloplastic material for orbital floor support (*p* = <0.04) (Table 3). Uptake of dental rehabilitation was sparse, and outcomes of speech and palatal healing was unreported.

**Table 2:** Characteristics of included studies

Scapular position	Specifics of orbital floor reconstruction	Author	Sample size	Months follow-up	Soft tissue obliteration	Maxillary access incision	Reported complications	Dental rehabilitation	VSP used
Vertical scapula for anterior maxillary wall & Green-stick osteotomy for horizontal fragment for orbital floor	Medial fragment used for orbital floor and lateral border for palate	Piazza^16^ 2017	10	27 (Mean)	Teres major	Not stated	1 Partial necrosis of TM 1 Dislocated orbital floor fragment	1 patient	No
		Clark et al.^17^ 2008	4	12 (Median)	4 Teres Major	1 Transfacial (Mustardé) 3 Weber-Ferguson	1 Ectropion	1 patient	No
	Distal tip of scapula used for orbital floor + recon plate for orbital rim	Haring et al.^18^ 2021	4	28 (Mean)	3 Not stated 1 Latissimus dorsi	Not stated	0	No	No
Vertical scapula for anterior maxillary wall	Additional scapula as bone graft for orbital floor	Clark et al.^17^ 2008	2	12 (Median)	1 Teres major 1 Latissimus dorsi	5 Weber-Ferguson 2 Lip-split	1 Ectropion	Not stated	No
Vertical scapula for anterior maxillary wall	Mesh (DiLeo and Choi) or plate (modest) for orbital floor	DiLeo et al.^19^ 2024	1	Not mentioned	Latissimus Dorsi	Unknown	1 Lagophthalmos	Not stated	No
		Modest et al.^20^ 2016	4	5.2 (Median	Latissimus Dorsi and serratus anterior	Unknown	1 Enophthalmos and ectropion	Not stated	Yes 1 case
		Choi^21^ et al. 2015	6	24 months	6 Latissimus Dorsi	Weber-Ferguson	1 diplopia	No	No
Horizontal scapula for palate	PSI or mesh for orbital floor	Cardín^a^ et al.^22^ 2024	11	40.64 ± 24.75 (Mean + SD)	8 Serratus anterior 2 Latissimus dorsi 1 TDAP	Not mentioned	Early 3 facial cellulitis 1 seroma Late (>2 weeks) 1 nasal obstruction 6 lacrimal duct obstruction 3 lagophthalmos 1 ectropion 3 exposed material 5 shallow vestibule 1 Bone flap failure 2 trismus	Yes in 4 patients	No
		Mertens^23^ 2012	1	4 years	Parascapular flap	Weber-Ferguson	Plate exposure Ectropion	Yes	Yes
	De-epithelialized skin paddle for orbital support	Granick^24^ 1990	1	Unknown	De-epithelialized parascapular flap	Weber-Ferguson	1 Enophthalmos with poor aesthetic result	Not stated	No
Horizontal scapula for orbital floor	De-epithelialized skin paddle for inferior support	Swartz^25^ 1986	2	Unknown	De-epithelialized parascapular flap	Unknown	None	Not stated	No
		Coleman et al.^26^ 1991	1	Unknown	De-epithelialized parascapular flap	Weber-Ferguson	Non stated	No	No
		Granick^24^ 1990	2	Unknown	De-epithelialized parascapular flap	Weber-Ferguson	None	Not stated	No

a Some patients had more than one late complication.

**Table 3:** Complications of various scapular orientations and orbital floor support.

Scapular position	Horizontal scapula for palate *n* = 13		Horizontal scapula for orbital floor *n* = 5	Vertical scapula for anterior maxillary wall *n* = 31			*p*-value
Orbital floor support	De-epithelialized skin paddle *n* = 1	Mesh/plate *n* = 12	Horizontal scapula *n* = 5	Green-stick osteotomized scapular fragment *n* = 18	Scapular bone graft *n* = 2	Mesh/plate *n* = 6	
Ocular complications	1	5	0	2	1	4	
Infections	0	3	0	0	0	0	
Plate/mesh complications	0	4	0	0	0	0	
N of patients with ≥1 complication	1	11^b^	0	2	1	4	
% complications^a^	100 %	92 %	0 %	11 %	50 %	26 %	≤0.001
Total % Complications	12 of 18 = 67 %			8 of 26 = 31 %			0.04

*n* = number of patients; Ocular complications: Enophthalmos, Diplopia, Ectropion, Lagophthalmos. Plate/mesh complications: Extrusions, Dislocation. ^a^Data analysis performed on number of patients with one or more complications ^b^one patient in Cardin et al. had both an ocular and mesh complication.

**Table 4:** Characteristics of included studies.

Scapular position	Specifics of orbital floor reconstruction	Author	Sample size	Months follow-up	Soft tissue obliteration	Maxillary access incision	Reported complications	Dental rehabilitation	VSP used
Horizontal scapula for palate	PSI or mesh for orbital floor	Cardín^a^ et al.^22^ 2024	11	40.64 ± 24.75 (Mean ± SD)	Eight serratus anterior Two latissimus dorsi One TDAP	Not mentioned	Early Three facial cellulitis One seroma Late (≥2 weeks) One nasal obstruction Six lacrimal duct obstruction Three lagophthalmos One ectropion Three exposed material Five shallow vestibule One bone flap failure Two trismus	Yes in Four patients	No
		Mertens^23^2012	1	4 years	Parascapular flap	Weber-Ferguson	Plate exposure Ectropion	Yes	Yes
	De-epithelialized skin paddle for orbital support	Granick^24^1990	1	Unknown	De-epithelialized parascapular flap	Weber-Ferguson	One enophthalmos with poor aesthetic result	Not stated	No
Horizontal scapula for orbital floor	De-epithelialized skin paddle for inferior support	Swartz^25^1986	2	Unknown	De-epithelialized parascapular flap	Unknown	None	Not stated	No
		Coleman et al.^26^1991	1	Unknown	De-epithelialized parascapular flap	Weber-Ferguson	Non stated	No	No
		Granick^24^1990	2	Unknown	De-epithelialized parascapular flap	Weber-Ferguson	None	Not stated	No

^a^Some patients had more than one late complication.

### Clinical case series—Descriptive data

**Figure 5:**
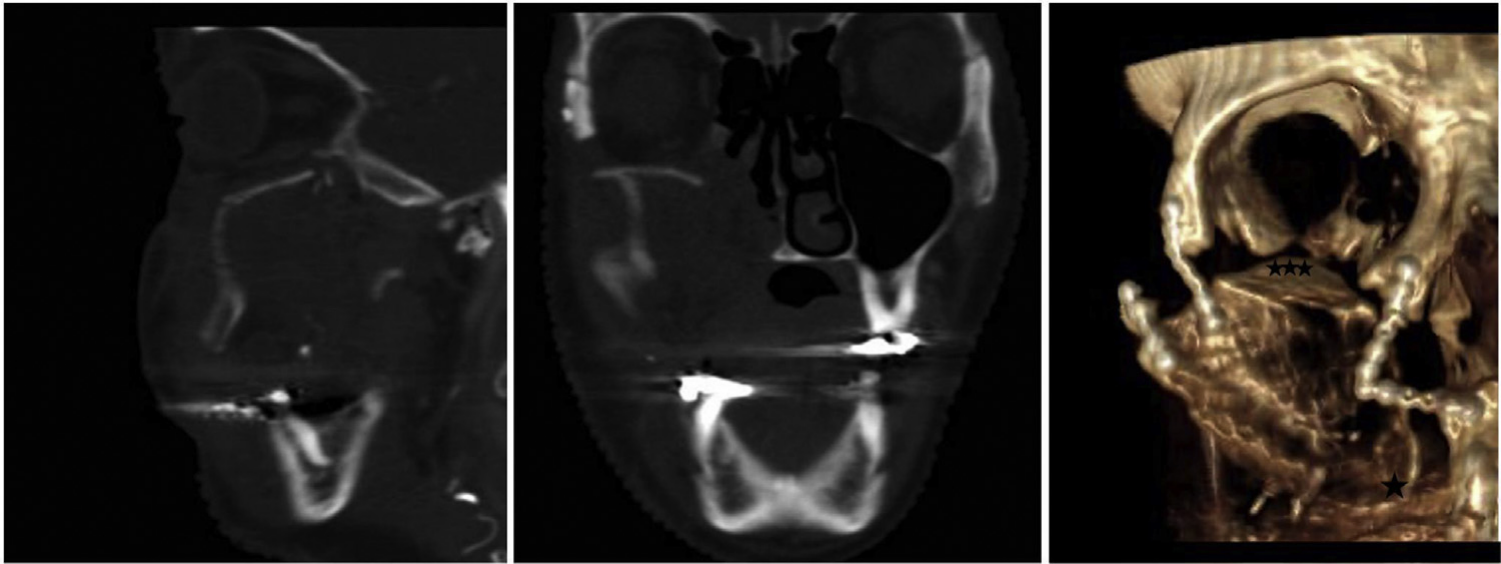
Vertical scapula with a green-stick osteotomy to reconstruct the orbital floor.

**Figure 6:**
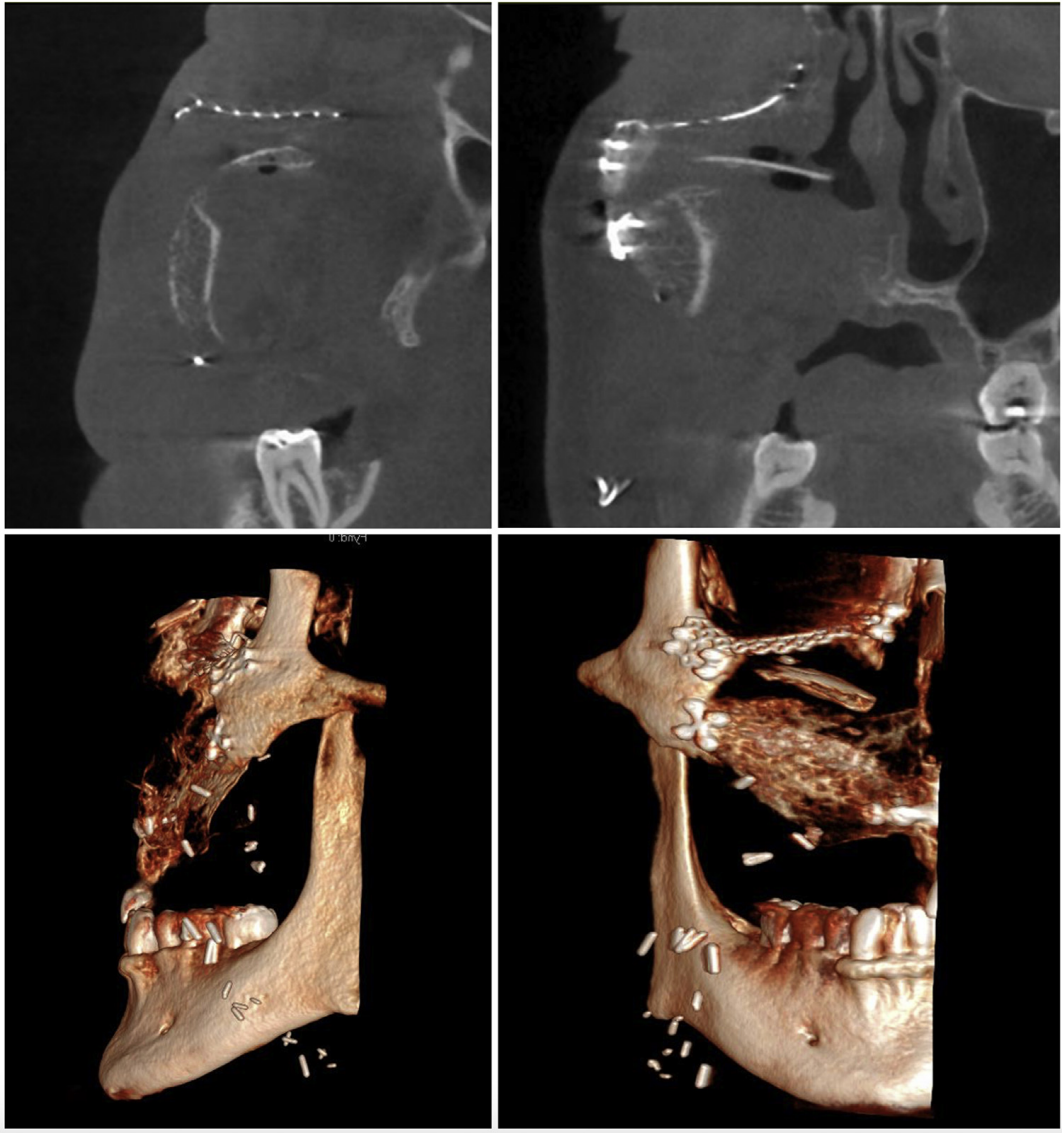
Vertical scapula with a greenstick osteotomy to reconstruct the floor with the addition of a patient specific implant for enhanced orbital support.

**Figure 7:**
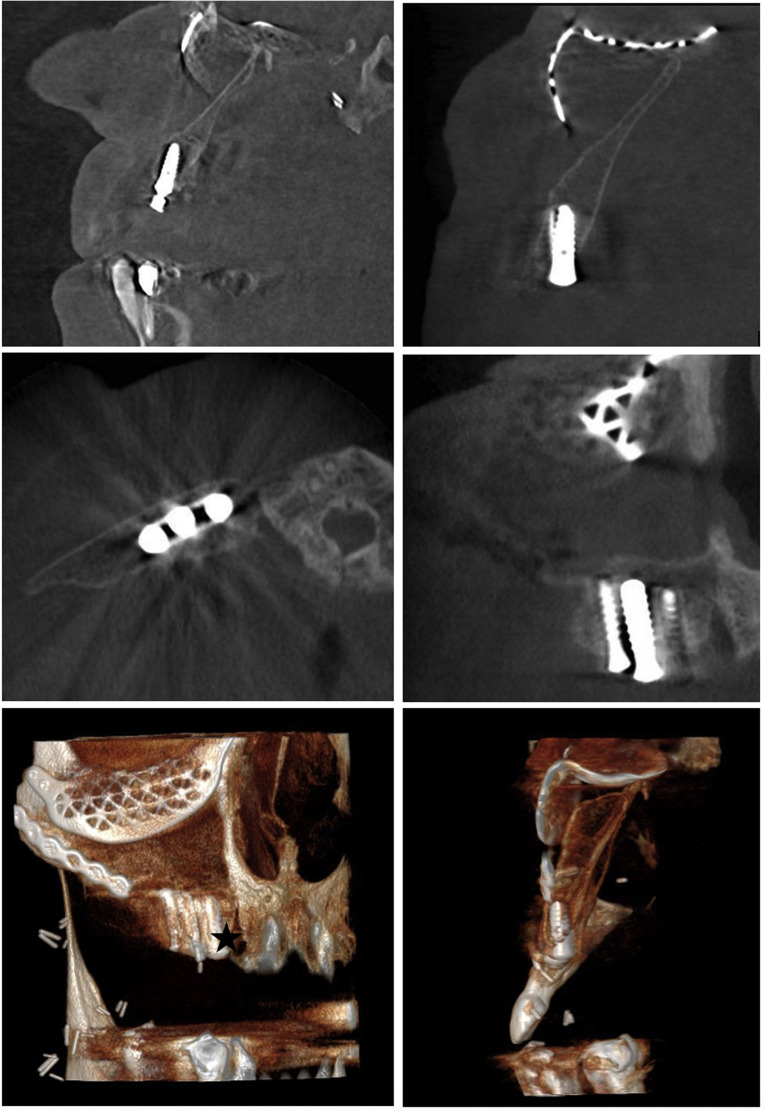
Vertical scapula with a green-stick osteotomy to reconstruct the orbital floor and a patient specific implant for enhanced support. Also showing three osseointegrated implants in the distal portion of the lateral scapula.

Four consecutive patients underwent total maxillectomies without orbital exenteration and were reconstructed with scapular free flaps based on the angular artery (Table 5). Exposure was achieved using the Weber-Ferguson incision with an infra-orbital extension. All reconstructions involved vertical scapular orientations with a green-stick osteotomy of the cranial medial portion of the scapula, folded and used to resurface the orbital floor (Figure 5). A patient specific implant or titanium mesh was added for orbital floor reinforcement in three of four cases (Figures 6 and 7). Flaps were harvested in a chimeric configuration with the latissimus dorsi or teres major muscle.^12^ Post-operative antibiotics included one dose of 1 g Metronidazole and three doses of 1,5 g Cefotaxime. Dental rehabilitation was completed in one patient. One patient was deferred due to ongoing pain and the need for a velopharyngeal flap to improve speech. Two patients passed away from metastatic disease within 2 years.

**Table 5:** Clinical case series—descriptive data.

Case Nr	Age-Sex	Diagnosis	Scapular position	Orbital floor addition	Bone lengths (cm)	Osteo-synthesis	Soft tissue lining	VSP	Recipient vessels	Maxillary access incision	Post-op RXT	Dental rehab
1	54-F	SCC	Anterior maxilla by vertical scapula Orbital floor by proximal medial fragment	None	6 (3.5 + 2.5)	Orbital plate + Mini-plates	LD	No	Facial vessels	WF	Yes	No
2	47-M	Myoepithelial Carcinoma		PSI	9 (6 + 3)	Orbital plate + Mini-plates	TM	Yes	Facial vessels	WF	Yes	No
3	65-M	Ameloblastoma		PSI	7 (5 + 2)	Orbital plate + Mini-plates	LD	Yes	Facial vessels	WF	No	Yes
4	38-F	AVM		Titanium mesh	5.5 (3 × 2.5)	Orbital plate + Mini-plates	TM	Yes	Facial vessels	WF	No	Yes

PSI (Patient specific implant), VSP (Virtual Surgical Planning), WF (Weber-Ferguson) SCC (Squamous cell carcinoma), AVM (Arteriovenous malformation), LD (Latissimus dorsi muscle), TM (Teres major muscle), RXT (Radiotherapy).

### Clinical case series—Complications

Infections were the most common complications, followed by orbital issues (ectropion, diplopia) (Table 6). Patient four experienced chronic pain, a malpositioned mesh requiring surgical correction and impaired speech requiring a velopharyngeal flap.

**Table 6:** Clinical case series—surgical complications.

Case Nr	Complication	Management
1	Abscess under flap Ectropion	Intravenous antibiotics Lateral tarsal slip
2	0	N/A
3	Abscess under flap	Antibiotics and surgical evacuation
4	Abscess under flap, Ectropion Malpositioned orbital plate causing diplopia, neuropathic pain, excessive tearing, speech impairment	Intravenous antibiotics Surgical correction of orbital plate, velopharyngeal flap

### Methodological quality

The rarity of publications resulted in finding case series or samples as case reports from larger studies. The levels of evidence were thereof low (ASPS Level IV or V). Similarly, the risk of bias was generally rated as medium or high risk across variables, with a mean overall risk of high (Table 7).

**Table 7:** ROBINS-I criteria for risk of bias assessment.

*Levels of Evidence for Therapeutic studies according to American Society of Plastic Surgeon. 1. Bias due to confounding, 2. Bias in classification of interventions, 3. Bias in selection of participants, 4. Bias due to deviations from intended interventions, 5. Bias due to missing data, 6. Bias in measurement of outcomes, 7. Bias in selection of the reported result.  Low Risk,  Moderate risk,  High risk. Two or more high risk outcomes = Overall High Risk Assessment.

## Discussion

Reconstruction of the midface after total maxillectomies without orbital exenteration demands a delicate balance between aesthetic restoration, orbital stability and functional rehabilitation. Achieving a balanced facial appearance with a correctly positioned eye and functioning vision, whilst managing the palato-maxillary projection and dento-alveolar restoration demands considerable surgical creativity and innovation in which the scapular free flap has become a popular option. This systematic review synthesized the evolution of scapular orientations and its impact on clinical outcomes. Our review found a higher rate of complications in horizontally orientated scapulas, especially when including non-vascularized material for orbital support.

### Orbital floor reconstruction

Ensuring a rigid and durable orbital floor is one of the main goals and challenges of total maxillectomies without orbital exenteration. An unproportionate orbital cavity and unstable orbital floor risks diplopia, enophthalmos and ectropion. Vascularized bone is preferred for a durable orbital floor reconstruction^1^^,^^9^ and using the scapular flap has grown in interest due to its flexibility in flap design and minimal donor site morbidity.^9^^,^^12^ Its use in total maxillectomies developed from an early experience of applying a horizontal orientation and focusing the reconstruction on the orbital floor or palate (with a primary alloplastic orbital floor reconstruction), to a vertical orientation with the aim of addressing both the palate and orbital floor in a “single flap” solution, with or without alloplastic material.

### Horizontal orientation: Anatomical congruence, but functional limitations

Early studies favored a horizontal orientation of the scapular free flap at either the orbital floor^13^, ^14^, ^15^ or palate^14^^,^^16^^,^^17^; using a de-epithelialized TDAP for palato-maxillary obliteration or orbital floor support, respectively. Although the horizontal scapula provides good anatomical congruence both at the palate^18^ and orbital floor,^19^ it falls short in addressing both areas simultaneously. Ocular complications were not reported in cases with horizontal scapulas at the orbital floor, however, using soft tissue as palato-maxillary obliteration is unreliable and risks midfacial collapse post-radiotherapy, secondarily affecting ocular positioning and function.^20^ Furthermore, soft tissue palatal resurfacing precludes osseointegrated dental rehabilitation. Adopting the opposite technique with a palatal horizontal scapula and soft tissue orbital floor support, proved unsurprisingly inadequate and was quickly abandoned.^14^ The palatal horizontal scapula was reintroduced by Cardin et al.^16^ and Mertens et al.,^17^ using alloplastic material as primary orbital floor support. Orbital floor instability with enophthalmos and diplopia however persisted, and infections and alloplastic extrusion occurred.

### Vertical orientation: Moving towards an integrated reconstruction

Shortcomings with achieving a multi-component reconstruction with the horizontal scapula shifted the surgical technique towards a vertical orientation, aiming for a “single flap” solution to reconstruct both the orbital floor and palate. Initially, vertical scapulas were used non-osteotomized and required alloplastic materials for orbital floor reconstruction, as seen in studies by DiLeo,^21^ Modest^22^ and Choi.^23^ Ocular complications, however, persisted.

### Osteotomized vertical scapular free flap: An all-vascularized palato-maxillary-orbital reconstruction

The design of the vertically orientated scapular free flap evolved to include a green-stick osteotomy, enabling a vascularized combined palato-maxillary-orbital reconstruction without alloplastic materials. By using either the distal scapular tip^24^ or (more commonly and morphologically more congruent to the native orbital floor) proximal medial scapula^25^^,^^26^ as the folded fragment, ocular complications from orbital floor instability (diplopia/enophthalmos) could be largely eliminated. However, Piazza et al.^26^ reported one case of dislocation of the orbital floor fragment. Lack of support at the posterior orbital cavity could cause tilting of the reconstruction, despite rigid fixation across the osteotomy. To address this, the authors used a hybrid approach in a clinical case series, combining a green-stick osteotomy with alloplastic material as secondary orbital floor support. Attempts to enhance the orbital floor did however not prevent complications, as infections, ocular issues and fragment mobility ensued; despite using virtual surgical planning for precision and thorough soft tissue obliteration.

### Palato-maxillary reconstruction & dental rehabilitation

Effective reconstruction of the palato-maxillary complex is vital for speech, swallowing, mastication and aesthetics. Resections spanning across the midline and/or into the soft palate result in poorer outcomes due to the impact on velopharyngeal competence.^8^^,^^27^ The scapular tip has been found to resemble the palate particularly well in a horizontal position,^18^ however, lacks midface support and bone thickness for osseointegrated implants. Despite this, dental rehabilitation was achieved in selected horizontal cases by Cardín^16^ and Mertens.^17^ The vertically orientated scapula with the lateral border distally placed is better suited for osseointegrated implants, as demonstrated by our case Nr 3 (Figure 7).^28^ Despite technical feasibility, completion of dental restoration is continually low due to for example, financial barriers, oncological progression and post-operative complications.^3^ Furthermore, whilst bulky muscle flaps are advocated for obliterative purposes, they can interfere with implant placements by contracting over time, blunting mucosal sulcii and retracting the lip, preventing adequate prosthetic coverage.^3^^,^^27^

### Controversies and the multifactorial nature of complications

Despite technical advancements of orbital floor support, complications with instability, infections and/or alloplastic exposures persist. The authors found least complications with the vertically orientated scapulae without alloplastic support^25^^,^^26^ with only one occurrence of dislocated orbital floor fragment and ectropion. Ectropion has been described as a common sequalae to the subciliary extension of the Weber-Ferguson exposure, for which reason a transconjunctival extension has been advocated.^2^^,^^29^ In the authors’ current practice, a vertical incision alone is utilized to mitigate this risk. The lack of posterior support in the orbital cavity is universal to any orbital floor reconstruction and risks posterior tilting and micromotion, particularly with mastication. Such instability can result in fluid accumulation and secondary infections.^5^^,^^26^^,^^30^ Enhancing stability by adopting a hybrid reconstruction with the folded scapula and alloplastic material failed to limit these complications in our clinical experience, as infections occurred in three of four cases. This is in contrast to other evidence supporting the use of non-vascularized material in combinations with vascularized reconstructions.^6^^,^^7^^,^^31^^,^^32^ Opinions are however divided, as alloplastic extrusion rates have been described in up to 23 % in maxillary reconstructions.^31^^,^^33^ but equally described as uncomplicated by Hanasono et al.^8^ and was by Schubert et al.^34^ showed to undergo full soft tissue integration despite a multicavity facial communication. A total maxillectomy results in a defect with direct communication between the bacterial flora of the naso- and oropharynx. Cavity separation and thorough soft tissue obliteration (often with muscle) is therefore critical. However, whilst full muscle epithelialization ensues, bony and/or non-vascularized reconstructions are potentially left exposed to the multi-bacterial flora during the initial post-operative period. Furthermore, contraction of the de-innervated muscle can lead to flap dehiscence and increased bacterial contamination of potentially exposed material. Overfilling the dead space is emphasized in order to counteract soft tissue shrinkage, fluid accumulation and secondary infections.^20^ A potential confounder is antibiotic protocol, which was inconsistently reported across studies. Whilst enhanced recovery protocols promote restrictive use (≤24 h),^35^ this may be inadequate for maxillectomy cases, which are subjected to potentially increased vulnerability due to multicavity bacterial contamination.

### Limitations

The rarity of total maxillectomies without orbital exenteration has resulted in a highly heterogeneous reconstructive landscape, making interpretation difficult.^7^ Studies consist of case reports and case series, increasing the risk of selection bias**,** confounding, and reporting bias. Current studies lack standardization of peri‑operative details and reporting of outcomes. Descriptions of scapular orientations are regularly unclear and terminology for alloplastic materials are used interchangeably. Antibiotic protocols are consistently lacking, but should be included in future publications as part of a standardized protocol of peri‑operative outcomes to enable conclusions on infection rates, particularly with alloplastic materials. Formal and objective assessment of velopharyngeal competence and speech is similarly currently overlooked, but should form a central part of post-operative assessment in order to gain full understanding of the functional impact of different scapular orientations.

Several potentially relevant studies were excluded due to insufficient details. Moya-Plana et al.^30^ reported on 84 reconstructions (38 with orbital floor involvement) but did not specify scapular orientation or stratify complications by technique. Similarly, Hasan et al.^36^ Triana et al.,^37^ Miles and Gilbert,^38^ Brown and Shaw^1^ did not detail the number of orbital floor reconstructions, scapular positioning, or associated complications. Ferri et al.^39^ describe both vertical and horizontal scapulae in maxillary reconstructions but did not clarify orbital or palatal scapular horizontal position, or present orientation-specific outcomes. The authors’ clinical case series, although uniform in technique, was limited by a small sample size (*n* = 4) and retrospective design. The role of alloplastic materials remains controversial as reported outcomes vary, and opinions differ on their safety and efficacy in maxillary reconstructions. Whilst some support their integration with vascularized flaps,^8^^,^^9^^,^^33^^,^^34^ others report high complication rates.^31^^,^^32^ At present, no definitive conclusions can be made regarding their routine use as primary or secondary orbital floor in total maxillectomies.

## Conclusion

Scapular free flap reconstructions for total maxillectomies without orbital exenteration have evolved significantly and adapted to complex multifaceted aesthetic and functional demands. However, achieving durable orbital stability remains a challenge. The current systematic review suggests that vertical scapular free flap reconstructions with a green-stick osteotomy offers the most comprehensive solution for a combined palato-maxillary-orbital reconstruction. However, ocular complications remain common in both vertical and horizontal orientations, and the role of alloplastic materials in enhancing orbital floor stability remains unsolved.

## Declaration of competing interest

None.
