# Erratum to ‘Horizontal versus vertical scapular free flap reconstructions in total maxillectomies without orbital exenteration—a systematic review and clinical case series’ [*JPRAS Open*. 2025;46:534–550]

**DOI:** 10.1016/j.jpra.2025.11.030

**Published:** 2025-11-29

**Authors:** Eleonora O.F. Dimovska, Francina W. Cobben, Andreas Thor, Andrés Rodriguez-Lorenzo

**Affiliations:** a Department of Plastic & Maxillofacial Surgery, Uppsala University Hospital, Uppsala, Sweden; b Department of Surgical Sciences, Uppsala University, Uppsala, Sweden; c Department of Plastic & Reconstructive Surgery, Leiden University Medical Centre, Leiden, The Netherlands

The publisher regrets that during the production process, some author corrections submitted at the proofing stage were inadvertently not incorporated into the final published version of this article. As a result, the published version contains errors that were not present in the corrected proofs.

In Table 2**,** the information for Choi et al. was incorrectly reported. The correct entry is: one patient and one latissimus dorsi flap, not six patients and six latissimus dorsi flaps.

The publisher would like to apologize for any inconvenience caused.

